# Feasibility, Safety, and Preliminary Effectiveness of a Home-Based Self-Managed High-Intensity Interval Training Program Offered to Long-Term Manual Wheelchair Users

**DOI:** 10.1155/2018/8209360

**Published:** 2018-05-17

**Authors:** Cindy Gauthier, Rachel Brosseau, Audrey L. Hicks, Dany H. Gagnon

**Affiliations:** ^1^School of Rehabilitation, Université de Montréal, Montreal, QC, Canada; ^2^Pathokinesiology Laboratory, Centre for Interdisciplinary Research in Rehabilitation of Greater Montreal, Centre Intégré Universitaire de Santé et Services Sociaux du Centre-Sud-de-l'Île-de-Montréal, Site Institut de Réadaptation Gingras-Lindsay-de-Montréal, Montreal, QC, Canada; ^3^Montreal Heart Institute, Montreal, QC, Canada; ^4^Department of Kinesiology, McMaster University, Hamilton, ON, Canada

## Abstract

**Objectives:**

To investigate and compare the feasibility, safety, and preliminary effectiveness of home-based self-managed manual wheelchair high-intensity interval training (HIIT) and moderate-intensity continuous training (MICT) programs.

**Methods:**

Eleven manual wheelchair users were randomly assigned to the HIIT (*n* = 6) or the MICT group (*n* = 5). Both six-week programs consisted of three 40-minute propulsion training sessions per week. The HIIT group alternated between 30 s high-intensity intervals and 60 s low-intensity intervals, whereas the MICT group maintained a constant moderate intensity. Cardiorespiratory fitness, upper limb strength, and shoulder pain were measured before and after the programs. Participants completed a questionnaire on the programs that explored general areas of feasibility.

**Results:**

The answers to the questionnaire demonstrated that both training programs were feasible in the community. No severe adverse events occurred, although some participants experienced increased shoulder pain during HIIT. Neither program yielded a significant change in cardiorespiratory fitness or upper limb strength. However, both groups reported moderate to significant subjective improvement.

**Conclusion:**

Home-based wheelchair HIIT appears feasible and safe although potential development of shoulder pain remains a concern and should be addressed with a future preventive shoulder exercise program. Some recommendations have been proposed for a larger study aiming to strengthen evidence regarding the feasibility, safety, and effectiveness of HIIT.

## 1. Introduction

A large proportion of individuals with a spinal cord injury (SCI) will need to learn to use a manual wheelchair as a means of mobility. Propelling a manual wheelchair requires good strength of the upper limbs (U/Ls) and trunk and good cardiorespiratory fitness [1–3]. However, most long-term manual wheelchair users (MWUs) with an SCI who live in the community adopt a sedentary lifestyle usually characterized by prolonged inactive sitting and limited physical activity [4, 5]. Hence, their cardiorespiratory fitness level is reduced which, in turn, could negatively affect their functional capacity and social participation while also increasing their risk of developing or exacerbating secondary cardiovascular and endocrine-metabolic complications [6, 7].

Long-term MWUs who want to engage in physical activity often face barriers such as a lack of accessible facilities and equipment or a lack of affordable transportation to access adapted facilities. Moreover, they often have difficulty developing and adapting their cardiorespiratory fitness training program due to their condition [8]. A simple way to overcome these barriers is to propose home-based self-managed training programs which eliminates the need for accessible facilities and transportation. MWUs can use their own manual wheelchair to participate in a cardiorespiratory fitness training program. Moreover, home-based training programs are recognized to be as effective as center-based programs while also having high adherence rates in cardiac rehabilitation [9]. However, only few studies have assessed the feasibility and effectiveness of home-based training programs among MWUs [10, 11].

Task-specific training protocols aiming at optimizing cardiorespiratory fitness among MWUs who propel their manual wheelchair have not been thoroughly studied. To date, the majority of studies on cardiorespiratory MWUs fitness programs have focused on arm-crank ergometers [12]. It is also worth noting that a task-specific [13] manual wheelchair cardiorespiratory training program could improve muscle strength (i.e., increased relative efficiency) as well as optimize wheelchair-related skills [14, 15]. These potential gains may minimize the risk of secondary U/Ls impairments and ultimately improve social participation among MWUs. Current exercise recommendations for individuals with an SCI predominantly suggest moderate to vigorous-intensity continuous training (MICT) exercises coupled with strengthening exercises [16, 17]. However, the focus of recent studies has shifted toward high-intensity interval training (HIIT). HIIT is expected to result in greater improvement in cardiorespiratory fitness than MICT [18–22]. HIIT is particularly interesting, since this training approach allows individuals to train at a higher intensity than MICT and for longer periods of time within a high-intensity range [23, 24], which we know is crucial for muscular (e.g., endurance, power) and cardiorespiratory adaptive changes [23, 24]. Overall, HIIT has been found to lead to better physiological adaptations than MICT in able-bodied and cardiac populations [22–28]. Only few studies have assessed HIIT among individuals with an SCI [29, 30] and only one has compared it to MICT [31]. However, exercise effects in able-bodied cannot be directly translated among individuals with an SCI since exercise is usually done with U/Ls by these while exercise is done by lower limbs by able-bodied individuals. Additional knowledge on the effects of HIIT among individuals with an SCI is needed, particularly since several activities of daily living require short intense bursts of exertion similar to those required in HIIT, such as ascending an access ramp in a manual wheelchair. Hence, offering a personalized home-based self-managed manual wheelchair HIIT program represents a promising option in order to provide a task-specific cardiorespiratory training program.

This pilot study investigated the feasibility, safety, and preliminary effectiveness of a home-based HIIT program compared to an MICT program among long-term MWUs with SCI living in the community. General areas of focus for feasibility [32] were explored in a questionnaire on the training programs. Safety of the home-based training programs was measured based on the number of adverse events. Finally, preliminary effectiveness on cardiorespiratory fitness and maximal isometric strength of key U/L muscle groups of each training program were measured and compared. It was hypothesized that both training programs are feasible and safe. It was also hypothesized that individuals in the HIIT group will experience greater cardiorespiratory fitness and U/L strength improvement in comparison with those in the MICT group.

## 2. Methods

### 2.1. Participants

A sample of 11 community dwelling long-term MWUs with an SCI was recruited for this study using a list of participants who previously participated in research project(s) and accepted to be contacted again. Each MWU underwent a clinical assessment performed by a physiotherapist to confirm eligibility for this study and to collect personal characteristics (age, type of injury, time since injury, and physical activity level [33]) and anthropometric parameters (weight and height) (Table 1). The inclusion criteria were to be between 18 and 65 years of age, to use a manual wheelchair as a primary means of mobility in the community and to reside within 75 km of the research center. Moreover, potential participants had to be independent in terms of basic wheelchair skills like propelling up ramps and negotiating turns/curves. Potential participants with medical contraindications to cardiorespiratory assessment and training according to American College of Sports Medicine (ACSM) standards [34] were excluded from the study. The Physical Activity Readiness Questionnaire for Everyone (PAR-Q+) [35] was completed by participants to ensure they could take part in a physical training program. Participants were excluded if they had any other associated condition or complication that could impede participation in the training or could be worsened by the training program. The Wheelchair User's Shoulder Pain Index (WUSPI) [36] was completed to screen for potential shoulder pain interference with the performance of various functional activities. Participants were excluded if their shoulder pain on the WUSPI was greater than 5/10 for question (5) (“pushing your manual wheelchair for 10 minutes or more?”) and question (6) (“pushing your manual wheelchair up ramps or inclines outdoors?”). The study was conducted directly in participants' homes. Ethical approval was obtained from the Research Ethics Committee of the CRIR (#CRIR-1068-0315). All participants reviewed and signed the informed consent form before entering the study.

### 2.2. Study Design

This study is an exploratory randomized, controlled, open-label trial with participants assigned to one of two groups: high-intensity interval training (HIIT) or moderate-intensity continuous training (MICT). Assessments were conducted for each participant before (*T*1) and after (*T*2) the home-based training programs.

### 2.3. Group Allocation

After the initial assessment, participants were randomly assigned to one of the two experimental groups: HIIT (*N* = 6) or MICT (*N* = 5). A blocked randomization method and a sequentially numbered, opaque, sealed envelope allocation concealment method were used.

### 2.4. Intervention

Participants completed the home-based training programs that incorporated three 40-minute training sessions per week over a period of 6 weeks. Before the training programs began, the physiotherapist first reviewed the attributes of the training program assigned to each participant. The physiotherapist also gave participants a paper copy of the Borg CR10 scale and explained how to use it to monitor exertion intensity [37]. The rate of perceived exertion (RPE) was used to monitor the exertion intensity since as a result of alteration of the autonomic system, individuals with an SCI above T6 have lower peak heart rate (HR_peak_) values [38]. Using a percentage of the HR_peak_ could have led to overestimated exercise intensity. Thereafter, each participant, with the help of the physiotherapist, planned out at least one training route where training sessions could be properly completed. Moreover, to ensure that participants had similar knowledge of the recommended manual wheelchair propulsion techniques, all participants watched a 5-minute tutorial video, highlighting the best propulsion techniques to preserve U/L integrity before starting their training program [39, 40].

The physiotherapist subsequently called participants on a weekly basis to ensure that the training program was running smoothly and being performed safely. They discussed the training route, barriers, and facilitators to training and adverse events as well as satisfaction with the assigned program. If there were any issues, the physiotherapist discussed them with the participant to find solutions (e.g., change the training route if the target exertion level was difficult to achieve on the initial route). The training schedule was modified if compliance was difficult to maintain due to constraints. Participants also completed a daily log book to describe the time spent exercising.

#### 2.4.1. High-Intensity Interval Training

Participants assigned to this group were asked to complete a 5-minute warm-up period at a rate of perceived exertion (RPE) between 2 (light) and 3 (moderate) on the Borg CR10 scale [37]. Participants were then asked to propel their wheelchair at high and low intensities during 30- and 60-second intervals, respectively, and to repeat this sequence 20 times over a total period of 30 minutes. During the 30-second high-intensity interval [41], participants needed to reach an RPE between 6 and 8 (very hard). Each high-intensity interval was followed by a 60-second low-intensity interval at an RPE between 1 (very light) and 2 (light). At the end of the training, there was a 5-minute cool-down period at an RPE between 2 and 3.

#### 2.4.2. Moderate-Intensity Continuous Training

Participants assigned to this group were also asked to complete a 5-minute warm-up period at an RPE between 2 and 3 on the Borg CR10 scale. Participants were then asked to propel their wheelchair for 30 minutes at a constant speed while maintaining an RPE between 4 (somewhat difficult) and 5 (difficult). At the end of the training, there was a 5-minute cool-down period at an RPE between 2 and 3.

### 2.5. Assessments and Outcome Measures

A clinical assessment was completed before (*T*1) and after (*T*2) the training programs. All tests and questionnaires were completed during a single assessment session done at each participant's home. For each participant, both assessments (*T*1 and *T*2) were performed at the same time of the day (±one hour) to limit the impact of the circadian rhythm on the cardiorespiratory outcome measures [42]. Moreover, participants were asked to avoid consuming caffeine or alcohol at least two hours prior to each test and were instructed to refrain from intense exercise the day before the test [43]. Upper limb strength assessment was performed first, followed by a rest period of approximately 30 minutes after which the cardiorespiratory fitness test was completed.

### 2.6. Satisfaction and Perceived Benefits

After completion of the training programs, participants completed a questionnaire to measure different aspects of feasibility of the training programs. This questionnaire had 17 items covering various domains: general satisfaction (i.e., acceptability (two items), feasibility of the training program (five items), perceived benefits for health (six items), perceived risks during training (three items), and motivation to remain physically active upon completion of the program (one item) (see Supplementary material (available here)). Participants were asked to rate their agreement with each sentence on a 7-point Likert scale ranging from 1 (complete disagreement) to 7 (complete agreement), except for questions on perceived benefits for health for which they were asked to rate their level of change using an Osgood's semantic differential scale ranging from 1 (significant deterioration) to 7 (significant improvement). For each question, the item assessed was judged as being favourable whenever the score was ≥5 (slight agreement), except for the perceived risk during training for which the threshold was set at ≤3/7 (slight disagreement).

#### 2.6.1. Feasibility

This study explored general areas of focus as proposed by Bowen et al. [32] for feasibility studies (i.e., acceptability, demand, implementation, practicality, and preliminary effectiveness). First, the acceptability represents the extent to which a program is judged suitable, satisfying or attractive. In this study, it is measured by the level of satisfaction (question (1)), the perceived appropriateness (question (2)), and the intent to continue (question (17)). The demand of the training programs represents the extent to which a program is likely to be used and it can be measured by the actual use (i.e., compliance rate). The implementation assesses if the program can be successfully put into practice by the participants in some defined, but not fully controlled contexts. Questions (3) to (6) of the questionnaire addressed implementation of the 6-week training programs. Practicality is the capacity to carry out the programs using the existing resources and context (i.e., the ability to reach the prescribed exercise intensity). Question (7) focused on the practicality of the cardiorespiratory fitness training programs directly in the community. Finally, the preliminary effectiveness has been measured by cardiorespiratory fitness testing and maximal isometric strength assessment. Moreover, subjective benefits following completion of both training programs were assessed by the questions (8) to (13) of the questionnaire.

A score of more than 5 (slight agreement) on 7 (complete agreement) on related questions was used to assess the feasibility of each aspect. For the subjective effectiveness, perceived benefits were confirmed whenever a participant rated their perceived improvements for a specific question at 5 (small improvements) or more on 7 (important improvements).

#### 2.6.2. Safety and Perceived Risks

The safety of each training program was determined by the absence of adverse events, including the absence of a significant increase in shoulder pain measured with the WUSPI questionnaire (i.e., >5.10 points of the total score which corresponds to the minimal detectable change [36]). Moreover, the perceived level of risk during the training programs was measured by questions (14) to (16).

### 2.7. Preliminary Effectiveness

#### 2.7.1. Cardiorespiratory Fitness Testing

All participants completed a maximal cardiorespiratory fitness test using a mechanically braked arm ergometer (Monark rehab trainer 881E, Vansbro, Sweden). Participants were first asked to cycle for a two-minute warm-up period. The test then started without resistance before being increased in 10 W increments every minute [44]. During the test, participants were asked to arm cycle at a minimum of 50 rpm and to continue to arm cycle until they reached volitional fatigue. The test was stopped when they were not able to maintain a 40-rpm cadence or exhibited abnormal cardiorespiratory measures according to the ACSM [34]. In order to measure cardiorespiratory responses, the participants were equipped with a portable respiratory gas analyzer device (Cosmed K4b^2^; Cosmed, Rome, Italy) which has been shown to be valid and reliable for measuring gas exchange during exercise [45]. Participants also wore a Polar® HR monitor (Polar FT4; Polar, Lachine, Canada) around their chest to measure their heart rate (HR). Participants were asked to rate their perceived U/L muscular exertion (RPE_muscle_) and their perceived cardiorespiratory exertion (RPE_cardio_) separately, using the Borg CR10 scale at the end of each test.

Peak values of VO_2_  (VO_2peak_) and HR (HR_peak_) were determined using the peak 20-second average of the test. The peak power output (PO_peak_) was defined as the greatest resistance reached during the test and was maintained for at least 15 seconds [44]. Exertion was considered to be maximal if participants attained RER > 1.1 or if a plateau in VO_2_ was reached (change < 2.1 mL/kg/min) with an increase in exercise intensity [46].

#### 2.7.2. Upper Limb Muscle Strength

Maximal isometric strength of the key U/L muscle groups (shoulder flexors/extensors, shoulder abductors/adductors, shoulder external/internal rotators, and elbow flexors/extensors) was performed prior to the cardiorespiratory test. Measurements were done on the dominant side using a handheld dynamometer (Medup®, Quebec, Canada). All muscle groups were tested in a supine position according to a standardized protocol to optimize participants' upper body stability and minimize compensation. For each muscle group tested, the lever's arm, which corresponds to the distance between the joint axis of the articulation being tested and the center of the location where the dynamometer head was applied, was measured. Two maximum voluntary contractions were recorded and if the two initial values differed by more than 20%, a third value was taken and the greatest value was selected as the outcome measure. Torque was calculated based on the lever arm measurement and was expressed in newton meters (Nm).

### 2.8. Statistical Analysis

A comparison of sociodemographic data, clinical data, outcome measures among both experimental groups (i.e., HIIT and MICT) at baseline, and outcome measures at *T*1 and *T*2 was performed using a Mann–Whitney *U* test with a significance level set at *p* < 0.05. The change between before (*T*1) and after (*T*2) the training programs [((*T*2 − *T*1)/*T*1)*∗*100] was computed for the main outcome measures and expressed as a percentage.

## 3. Results

### 3.1. Participants

Eleven individuals with an SCI were recruited to participate in this study; six participants were randomized into the HIIT group and five into the MICT group. There was no statistical difference in participant characteristics between the two groups (Table 1).

### 3.2. Recruitment Flow

**Figure 1:**
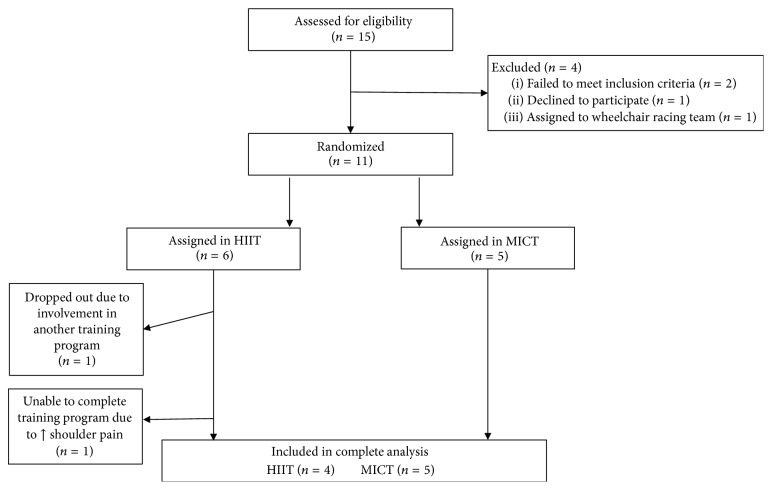
Participant recruitment flow diagram.

Two participants in the HIIT group failed to complete all aspects of the study: one participant dropped out at the beginning of the project because he was involved in another training program and one participant dropped out due to a significant increase in shoulder pain after six training sessions (+19.2/150 on the WUSPI). Four participants successfully completed the HIIT program and five completed the MICT program. The dropout rate was 33% and 0% for the HIIT and MICT groups, respectively. The participant recruitment flow diagram is presented in Figure 1.

### 3.3. Feasibility

**Figure 2:**
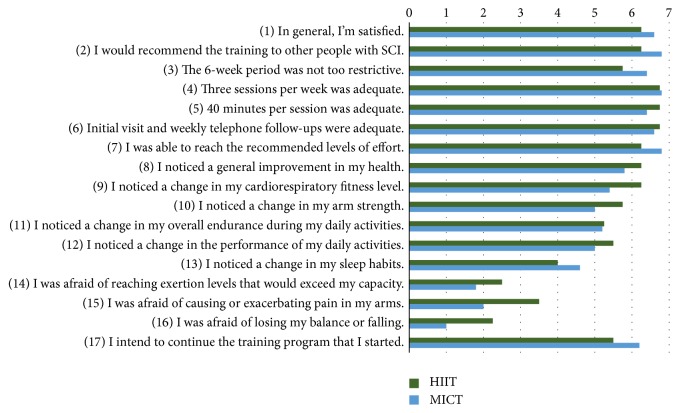
Mean score for each of the questions included in the questionnaire completed at the end of the training programs.

The feasibility of the training programs has been assessed, in some parts, by a questionnaire on satisfaction and perceived benefits. A summary of the results of the questionnaire is illustrated in Figure 2. For all questions, no statistically significant difference was found between groups.

#### 3.3.1. Acceptability

Concerning the acceptability of both training programs, all participants were generally satisfied (question (1)) with their training program except one in the HIIT group who was neutral (i.e., 4/7 on the Likert scale). Moreover, all participants in both groups would recommend their training program to other people with an SCI (question (2)) and all participants, except one in the HIIT group, intended to continue their training program (question (17)).

#### 3.3.2. Demand

Compliance for both training programs was very high with 86.11% for the HIIT program and 97.78% for the MICT program. To be considered feasible, a threshold of compliance rate of at least >75% (i.e., >13 completed sessions/18 planned sessions) was used [47]. Overall, participants in the MICT group completed more training sessions (17.6 ± 1.7 sessions; range 16–20) than the participants in the HIIT group (15.5 ± 2.1 sessions; range: 13–18). One of the participants in the MICT group initially had difficulty training continuously for 30 minutes; therefore, he divided his training sessions into two 10–20-minute sessions for the first two weeks.

#### 3.3.3. Implementation

All participants considered that the training programs proposed were adequate considering their availability, except for one, who found the six-week program slightly restrictive (i.e., 3/7 on the Likert scale). The completion of three sessions of 40 minutes was considered feasible by all participants.

#### 3.3.4. Practicality

Participants in both groups reported that the training programs were feasible in their community as they were able to reach the prescribed exercise intensity. Nonetheless, some individuals in the HIIT group mentioned that it was sometimes difficult to find an appropriate road, path, or walkway to train at high-intensity and, even more importantly, to minimize collision risk exposure.

#### 3.3.5. Preliminary Effectiveness

The results of the cardiorespiratory fitness testing and maximal isometric strength assessment will be presented in a next section. Concerning the perceived effectiveness of the training programs, there was a tendency for the HIIT group to perceive their training program as more beneficial, even if there was no statistical difference between both groups for their responses to the questionnaire. Indeed, participants in the HIIT reported mostly important improvements (i.e., 7/7 on the Osgood's semantic differential scale) for all questions except one participant who reported no change for all questions on perceived benefits. In the MICT group, the improvements were considered small (5/7) to moderate (6/7). All participants in both groups, except one, had no change in their sleep habits.

### 3.4. Safety

All participants successfully completed the maximal cardiorespiratory fitness test. Moreover, no adverse events occurred during the maximal cardiorespiratory fitness test or the training programs. However, one dropout in the HIIT group was due to the development of significant shoulder pain (WUSPI score = 33.8/150) over the course of the training program. This participant had the highest score on the WUSPI at *T*1 (i.e., initial WUSPI score = 14.6/150). The pain gradually increased over the first six training sessions and reached values ≥ 5/10 for questions (5) (i.e., pushing your manual wheelchair for 10 minutes or more?) and (6) (i.e., pushing your manual wheelchair up ramps or inclines outdoors?) and the total score increased for more than 5.1. The decision to stop the training program was then made upon mutual agreement between the physiotherapist and the participant. However, the pain was mild in most functional activities, except for manual wheelchair propulsion and for work-related tasks (i.e., question (12)) during which the pain score was higher than 5/10. Since the overall pain was moderate (i.e., 25.6/150), this participant decided to continue to train using the MICT program. His shoulder pain decreased at the end of the six-week period compared to the baseline level (initial WUSPI score = 14.6/150) at *T*1 and WUSPI score at *T*2 (i.e., 6.65/150). This participant's results were not considered in the statistical analysis. At the end of the HIIT program, two participants reported a significant increase in their total WUPSI score (+10.0/150 and +5.3/150), while no significant increase in shoulder pain occurred in the MICT group. There was no significant difference in baseline WUSPI scores between the groups (HIIT = 6.0 ± 2.3; range: 3.2–8.7 and MICT = 8.0 ± 5.5; range: 1.7–13.7, *p* = 0.730). Shoulder pain increased by 2.62 ± 6.23; range: −3.85–10 points on the WUSPI in the HIIT group and decreased by 0.62 ± 3.81; range: −6.4–4.1 points in the MICT group.

Concerning the perceived risks during the training programs, participants in the HIIT group judged their training as being riskier than the participants in the MICT group. Indeed, two participants were afraid to have initial or increase U/L pain; one was afraid to exceed his capacity, and one was afraid to lose balance during training. In the MICT group, one participant was afraid to have U/L pain.

### 3.5. Preliminary Effectiveness

#### 3.5.1. Change in Cardiorespiratory Fitness

**Figure 3:**
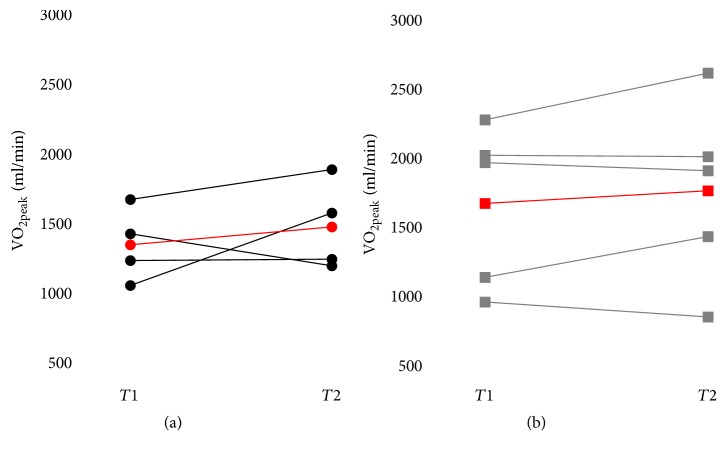
VO_2peak_ values before (*T*1) and after (*T*2) the training program for each participant in the HIIT group (a) and the MICT group (b). Red lines represent the mean changes for each group.

All changes in cardiorespiratory fitness outcome measures are presented in Table 2. There was no statistically significant between-group difference for all baseline cardiorespiratory outcome measures (*p* = 0.063–1.000) or for relative change (expressed as a %) (*p* = 0.413–0.905). No statistically significant within-group difference was found between *T*1 and *T*2 for both groups. Moreover, VO_2peak_ values were plotted on graphs for each participant and lines were drawn between the data before and after the training to highlight the directionality of the effects on each participant in both groups (Figure 3). Considering that the minimal detectable change for VO_2peak_ found in the literature is between 22% and 29% among manual wheelchair users [48, 49], only one participant was judged to have improved in the HIIT (i.e., +50.47%) whereas the other participants reached almost similar values (i.e., +13.08%, 0.83% and −16.34%). Similarly, in the MICT group, VO_2peak_ values were judged to have improved for one participant (i.e., +26.53%) whereas the other participants reached almost similar values (i.e., +15.04%, −3.01%, −0.49% and −11.61%).

#### 3.5.2. Upper Limb Muscle Strength Adaptation

All changes in U/L strength outcome measures for the right U/L (i.e., dominant side) are presented in Table 3. There was no statistically significant between-group absolute difference at *T*1 (*p* = 0.063–0.730) for all outcome measures. For relative difference (expressed as a %), no statistically significant between-group difference was found (*p* = 0.190–0.905), except for a change in shoulder external rotation strength (*p* = 0.016). In the HIIT group, one participant had an overall improvement (i.e., +12.63%) in U/L muscle strength and three participants maintained their strength level (i.e., +3.61%, −1.14%, and −3.79%). In the MICT group, four participants increased their mean U/L muscle strength (i.e., +15.84%–23.51%) whereas one participant maintained the same level of strength (i.e., +7.49%).

## 4. Discussion

The main objective of this pilot study was to investigate the feasibility, safety, and preliminary effectiveness of a home-based HIIT manual wheelchair program offered to long-term MWUs and to compare it to a MICT program. The results of this study suggest that an HIIT program appears feasible and safe and has comparable effects on most cardiorespiratory fitness and U/L muscle strength values versus an MICT program. However, special attention should be paid to exercise and shoulder pain when beginning an HIIT program, especially in individuals with prior shoulder pain. In fact, each training program should be personalized in type (HIIT, MICT, or a combination of both), duration, intensity, training route, and so on to allow for suitable progression and prevent adverse events such as an increase in shoulder pain. In addition, weekly follow-up by a health care professional may help to ensure that the training program is suitable for each individual.

### 4.1. Feasibility

Both training programs appear feasible since they were judged to be acceptable, practicable implementable, and effective. Indeed, all participants completed at least 75% of the planned training sessions. There was a small, insignificant difference in compliance between the groups. This difference may be explained by the fact that all participants in the HIIT group were already participating in three or more physical activity sessions per week. This may have made it more difficult for them to add three training sessions per week into their schedule. Some of them were involved in team sports and could not miss their practice sessions. Some exercise studies among an SCI population have suggested that training programs that include two training sessions per week should be favoured over three sessions to increase compliance, especially with a long-term training program [50, 51]. In fact, the Canadian physical activity guidelines for adults with an SCI [17, 52] recommend two 20-minute sessions of moderate to high-intensity cardiorespiratory exercise for general fitness. However, these guidelines suggest three 30-minute sessions of moderate to high-intensity aerobic exercise to anticipate cardiometabolic health benefits [17]. To this effect, studies using a frequency of two sessions per week have only reported improvements after a longer training period (>12 weeks) [50, 53], while programs incorporating three sessions per week have reported improvements after only six weeks [12, 29, 30]. Therefore, if improvements in cardiorespiratory fitness are sought in a short period of time, a frequency of three training sessions per week may be necessary. In the long term, however, a training frequency reduction to two sessions per week may enhance compliance with the exercise program and prevent musculoskeletal secondary impairments.

All participants felt that 40-minute training sessions were adequate. Participants in both groups were satisfied with the target workload intensity and were able to achieve it by propelling in the community. However, some adjustments in the road traveled during their training were required during the first few sessions for some participants in the HIIT group in order to reach the target intensity, especially on level ground. Environments with slopes or high rolling resistance surfaces (e.g., carpeted floor, grass) were preferred in order to attain the high-intensity exercise level. Moreover, certain participants reported that propelling directly on certain streets or sidewalks was difficult at times when the cross-slope was too pronounced and the propulsion technique needed to be modified and became asymmetric to a great extent. In these types of situations, frequent direction changes were needed to prevent participants from developing isolated U/L fatigue or discomfort. Participants who trained on a bicycle path enjoyed the program more because they felt they had a clean, safe road to propel on. Participants living in the countryside felt less safe when they had to propel directly on the paved road because of damaged asphalt, rocks, and cars passing closely next to them. Finally, participants reported that the weekly telephone follow-up helped to maintain their motivation and provided them an opportunity to answer all possible concerns about their training.

### 4.2. Safety

Both training programs were considered to be safe since no severe adverse events occurred. However, precaution should be taken when beginning an HIIT program since the increased mechanical load and muscular demand documented during propulsion can lead to the development or exacerbation of U/L pain, especially at the shoulder [54]. At the end of the HIIT program, two participants reported a significant increase in their total WUPSI score (+10.0/150 and +5.3/150), and one participant dropped out because of shoulder pain (+19.2/150). Except for the latter, the increase in shoulder pain did not specifically manifest itself during manual wheelchair propulsion or during a specific functional activity. In fact, the participant reported small increases in shoulder pain while performing a few functional activities which may have been due to late-onset muscle soreness or fatigue. Moreover, the participant who dropped out of the HIIT group because of shoulder pain was the one with the highest WUSPI score at baseline and was known for episodic increases in shoulder pain due to rotator cuff weakness. The fact that this participant experienced a decrease in shoulder pain after exercising at moderate intensity indicates that the HIIT program was probably too strenuous in the presence of shoulder pain and weakness. Since propelling puts a considerable load on the rotator cuff [54], posterior shoulder and rotator cuff muscle-strengthening exercises should be added to the training program. Moreover, anterior shoulder muscle stretches are recommended in order to prevent shoulder pain [55]. Given that the Canadian physical activity guidelines for adults with an SCI recommend two sessions of strengthening and stretching per week, these should be added to cardiorespiratory training program [52].

### 4.3. Preliminary Effectiveness

In both groups, one participant improved their VO_2peak_ by more than the minimal detectable change reported in the literature (i.e., between 22% and 29%) [48, 49] while the other participants had no significant change. It is worth noting that little information on reliability and minimal detectable change of VO_2peak_ tests among individuals with an SCI is available in the literature so the interpretation of the present results is limited. The participant in the HIIT group who improved the most in cardiorespiratory fitness was already training on a sports team with a coach and could not miss practice sessions. Therefore, he added the cardiorespiratory fitness training to his weekly exercise training program. The other active participants, except one who played basketball, were training by themselves, so most of them did not increase their amount of training during the study since they replaced their usual training sessions by the HIIT sessions. It seems that six weeks of the proposed HIIT program alone may not be strenuous and long enough to improve VO_2peak_ in MWUs who are already active, but if it is added to another training program, it can improve cardiorespiratory fitness. In the MICT group, the participant who improved his fitness level was the most enthusiastic about the training program during the weekly follow-up while his compliance rate was the same as the other participants. This participant also reported being assiduous in his training sessions and consistently following the instructions and intensity of the prescribed exercise. The enjoyment and motivation in the training program expressed by this participant may have reduced his perceived effort for a given exercise intensity and increased his exercise endurance during training [56].

Even if some participants improved, no statistically significant change in the group mean was found at the end of both six-week training programs. It is possible that with a larger sample statistically significant changes could have been observed. There is evidence to suggest that a training program incorporating three 20- to 60-minute moderate intensity exercise sessions per week for at least six weeks is effective in improving cardiorespiratory fitness in individuals with an SCI [12, 57]. It is worth noting that the exercise training programs in those studies were supervised by a healthcare professional so that the intensity of the exercise could be well controlled. However, since the current study involved home-based interventions, training sessions were not directly supervised and the exercise intensity was managed by the participant's perception of exertion using the Borg CR10 scale. Using this subjective scale could have altered the exercise workload. Indeed, while RPE monitoring allows a gradual and personalized workload progression since the individuals can gage their workload intensity according to their RPE, there it is possible that perceptions of exertion can be over- or underestimated [56]. In fact, RPE is recognized to be affected by psychological factors including cognitive factors such as self-efficacy, motivation, interoceptive feedback as physical discomfort, and perception of fatigue [56, 58]. Moreover, RPE and exercise tolerance are more influenced by interoceptive feedback than cognitive factors during high-intensity exercise than low-intensity exercise. This is because during high-intensity exercise the sensation of discomfort is more noticeable [59]. Some cognitive strategies like distraction techniques and motivation interventions can be used to increase high-intensity exercise tolerance and reduce RPE especially among inactive individuals [56]. On the other hand, considering the fact that HIIT is more aversive than MICT [60], it has been found to be more than or as enjoyable as MICT in individuals with an SCI and active and inactive individuals [60–63] and to elicit the same compliance [63]. Finally, despite the lack of statistically significant change in the main outcome measures selected, most participants still reported considerable and significant subjective improvements in their general health, including cardiorespiratory fitness.

In terms of strength improvements, one participant improved in general U/L muscle strength. Three participants had no change in general U/L muscle strength in the HIIT group, while four participants increased in the MICT group and one had no change. However, the decreases in strength in the HIIT group were very small and insignificant. It was expected that the high-intensity period included in the HIIT program would allow exertion sufficient to increase muscle strength. It was also expected that six weeks of training would be enough to improve muscular strength since a previous study reported improvement in shoulder flexor and extensor strength after only five weeks of wheelchair ergometer training in individuals with SCI [64]. However, training on a wheelchair ergometer allows resistance to be well controlled and progressively increased as muscle strength increases. During community wheeling, however, the only way to increase resistance is to wheel over high-resistance surfaces or up a hill or an access ramp. In many cases, participants in the current study elected to go faster to reach the required exercise intensity because they did not have access to a hill or ramp and had no control over the surface resistance. However, propelling faster is more likely to improve muscle power than strength [65]. In this study, only isometric maximal muscle strength via maximal voluntary contractions, not power, was assessed using a handheld dynamometer. In future studies on home-based manual wheelchair training programs, it would also be useful to assess muscle power to better investigate the effectiveness of the training programs.

Finally, the small degree of change in cardiorespiratory fitness and strength outcomes could be explained by the fact that almost all participants included in this study were already physically active. The two participants in the MICT group who did not exercise regularly showed the biggest increases in VO_2peak_ (i.e., 26.53% and 15.04%) and the ones who were the least active also had one of the greatest improvements in U/L muscular strength (i.e., +20.16% on average). Hence, future studies should target sedentary MWUs with an SCI since they are the ones that might benefit the most from a cardiorespiratory fitness program.

### 4.4. Future Studies

This study was a pilot study to assess feasibility, safety, and preliminary effectiveness of a home-based HIIT program among MWUs with an SCI since very few studies have examined HIIT programs in this population [31, 61] and no studies have assessed home-based HIIT programs. In this study, both training programs had the same workload per session so that the programs could be compared. However, since it was an exploratory study and safety was uncertain, the high-intensity periods were not set at maximal intensity but rather at near maximum (i.e., RPE on Borg CR10 scale between 6/10 and 8/10). Moreover, the low-intensity periods were two times longer than the high-intensity periods. Considering those facts, the total time duration of HIIT sessions had to match that of the MICT sessions. However, one of the advantages of HIIT programs is that they require less time to obtain similar or even better results than MICT programs [31, 66]. Future studies could propose HIIT programs with shorter training sessions and higher high-intensity periods. Moreover, this study did not report any significant change in cardiorespiratory fitness or U/L muscle strength after the six-week home-based training programs, while some studies reported significant changes after six-week supervised training programs [12, 29, 57]. It would be very interesting to compare the effectiveness of home-based training programs and supervised training programs among MWUs in future studies. Lastly, future studies could have an adaptive trial design in order to establish the criteria for beginning an HIIT program, such as the level of shoulder pain or key shoulder muscle strength.

### 4.5. Limitation

The principal limitation of this study was the small sample size in both groups that drastically reduced the statistical power. There was also a selection bias since most participants were already physically active and were more likely to be fully engaged and motivated in a cardiorespiratory fitness program. Moreover, the amount of exercise performed by each participant was different in time and intensity since they were asked to continue their usual physical activities. In future studies, nonexercising participants should be favoured or participants should be asked to stop their physical activities. Since exercise intensity was self-monitored by the participant using the Borg CR10 scale, there may have been differences in intensity between participants in the same program depending on their motivation. Finally, no non-exercise control group was used and it may be needed in future studies to better identify training effects.

## 5. Conclusion

This study suggests that a home-based HIIT cardiorespiratory fitness training in manual wheelchair consisting of three training sessions of 40 minutes is feasible and safe for MWUs, although special attention should be given to shoulder pain. However, future studies are needed to confirm safety and effectiveness. Following this study, some recommendations for future studies are proposed:

*Methodological Recommendations*

- Compare home-based training programs and supervised training programs;
- Add a non-exercise control group;
- Validate the use of RPE to assess the intensity;
- Measure muscle power;
- Target sedentary or minimally active individuals;
- Employ an adaptive trial design.

*Training Recommendations*

- Add sessions of strengthening and stretching to the training program;
- Longer training program;
- Shorter training sessions and higher high-intensity periods;
- Weekly follow-up to ensure appropriate progression and safety and to maintain motivation;
- Offer guidance on the selection of the training road;
- Use cognitive strategies like distraction techniques and motivation interventions.

## Figures and Tables

**Table 1:** Description of participants.

Participants	Sex	Age (years)	Height (m)	Weight (kg)	Time since injury (years)	BMI (kg/cm^2^)	SCI level	AIS	Level of physical activity
HIIT group									
1	F	34.3	1.75	70.8	5.1	23.1	T6	C	c
2	M	32.9	1.93	84.9	9.0	22.8	T6	A	c
3	M	37.8	1.8	65.0	8.7	20.1	T10	B	c
4	M	30.6	1.8	66.1	1.3	20.4	C7	B	c

*Mean ± SD (range)*		*33.9 ± 3.0 (30.6–37.8)*	*1.8 ± 0.1 (1.8–1.9)*	*71.7 ± 9.2 (65.0–84.9)*	*6.0 ± 3.6 (1.3–9.0)*	*21.6 ± 1.6 (20.1–23.1)*			

LMCT group									
1	M	22.7	1.90	73.0	2.8	20.2	C6	A	c
2	M	58.6	1.75	97.6	22.8	31.9	T10	A	c
3	M	63.9	1.9	95.6	16.6	26.5	T10	A	b
4	M	26.5	1.83	78.1	5.4	23.3	T11	C	a
5	M	44.1	1.76	105.00	3.8	33.9	T11	A	c

*Mean ± SD (range)*		*43.2 ± 18.5 (22.7–63.9)*	*1.8 ± 0.1 (1.8–1.9)*	*89.9 ± 13.6 (73.0–105.0)*	*11.5 ± 10.3 (2.8–22.8)*	*27.2 ± 5.7 (20.2–33.9)*			

Drop out									
1	M	26.3	1.83	72.5	2.0	21.6	C7	B	c
2	F	39.7	1.65	73.4	4.8	27.0	T2	A	c

*Mean ± SD (range)*		*33.0 (26.3–39.7)*	*1.7 (1.7-1.8)*	*73.0 (72.5–73.4)*	*3.4 (2.0–4.8)*	*24.3 (21.6–27.0)*			

(a) Physically inactive or sedentary (less than one exercise session (≥20 minutes) per week), (b) irregularly or moderately active (between one and two exercise sessions per week), and (c) active (three or more exercise sessions per week).

**Table 2:** Cardiorespiratory fitness outcome measures before (*T*1) and after (*T*2) the training programs.

	HIIT				LMCT				Dropout
	*T*1	*T*2	Change (%)	*p*	*T*1	*T*2	Change (%)	*p*	*T*1
VO_2peak_ (ml/min)	1325.9 ± 264.9	1455.4 ± 322.4	12.0 ± 28.3%	0.625	1650.0 ± 584.9	1741.6 ± 661.8	5.3 ± 15.3%	0.813	940.3
	(1033.6–1651.4)	(1176.13–1867.4)	(−16.3–50.5%)		(937.0–2254.7)	(828.2–2594.0)	(−11.6–26.5%)		(835.0–1045.7)
VO_2peak_ (ml/min)	19.5 ± 0.7	20.4 ± 3.9	5.4 ± 23.6%	0.875	18.5 ± 6.8	18.9 ± 8.4	2.4 ± 18.2%	1.000	9.6
	(18.5–20.1)	(15.8–23.9)	(−21.4–29.6%)		(11.6–28.9)	(11.8–33.3)	(−18.9–26.5%)		(7.7–11.6)
HR_peak_ (bpm)	161.5 ± 26.6	153.0 ± 15.7	−4.2 ± 10.3%	0.500	132.4 ± 42.0	138.8 ± 33.3	8.2 ± 22.2%	0.875	105.0
	(127.0–183.0)	(136.0–174.0)	(−17.6–7.1%)		(87.0–179.0)	(86.0–175.0)	(−4.3–47.8%)		(90.0–120.0)
PO_peak_ (W)	80.0 ± 18.3	80.0 ± 14.1	1.0 ± 11.0%	1.000	80.0 ± 25.5	82.0 ± 27.7	1.8 ± 14.3%	1.000	50.0
	(60.0–100.0)	(70.0–100.0)	(−11.0–17.0%)		(50.0–110.0)	(40.0–110.0)	(−20.0–16.7%)		(40.0–60.0)
RPE_muscu_ (/10)	9.75 ± 0.5	10.0 ± 0.0	3.0 ± 6.0	1.000	8.2 ± 1.8	8.8 ± 1.1	9.5 ± 15.0%	1.000	9.5
	(9.0–10.0)	(10.0)	(0–11%)		(6.0–10.0)	(7.0–10.0)	(−10.0–28.6%)		(9-10)
RPE_cardio_ (/10)	7.75 ± 2.9	7.75 ± 3.3	−3.0 ± 16	1.000	7.8 ± 3.0	7.2 ± 3.3	−9.6 ± 25.4%	1.000	8.5
	(4.0–10.0)	(3.0–10.0)	(−25.0–14%)		(3.0–10.0)	(2.0–10.0)	(−40.0–14.3)		(8-9)

**Table 3:** Right upper limb strength outcome measures before (*T*1) and after (*T*2) the training programs.

	HIIT				MICT				Dropout
	*T*1	*T*2	Change (%)	*p*	*T*1	*T*2	Change (%)	*p*	
Shoulder									
Flexors	49.9 ± 3.5	51.5 ± 6.4	10.2 ± 15.4%	0.375	62.4 ± 15.3	66.2 ± 17.6	6.2 ± 12.4%	0.438	43.3
	(43.5–51.4)	(46.0–60.7)	(−10.4–26.9%)		(50.0–82.2)	(47.4–91.8)	(−6.8–24.9)		(40.1–46.5)
Extensors	64.5 ± 15.9	59.6 ± 15.6	−7.25 ± 13.7%	0.375	87.7 ± 25.0	87.2 ± 25.5	12.2 ± 20.8%	0.438	53.2
	(43.1–81.3)	(38.3–72.8)	(−16.4–13.1%)		(43.8–104.9)	(52.2–118.7)	(−21.5–35.6%)		(52.7–53.6)
Abductors	46.8 ± 12.5	51.0 ± 16.6	7.6 ± 10.2%	0.250	56.1 ± 18.6	70.9 ± 15.1	33.0 ± 37.9%	0.188	40.4
	(33.7–59.0)	(31.1–67.1)	(−7.6–13.7%)		(40.9–87.1)	(48.3–88.1)	(−14.5–89.9%)		(29.4–51.3)
Adductors	69.5 ± 5.0	66.6 ± 7.2	−3.4 ± 15.8%	0.625	73.5 ± 28.2	71.4 ± 29.1	−1.7 ± 35.4%	1.000	40.1
	(64.3–75.6)	(57.6–73.8)	(−19.2–10.2%)		(45.4–113.0)	(23.4–99.5)	(−48.5–50.1%)		(34.8–45.5)
Internal rotators	49.2 ± 20.7	51.7 ± 25.0	3.8 ± 12.0%	0.875	53.2 ± 22.6	62.7 ± 17.8	30.9 ± 43.8%	0.188	36.7
	(30.6–69.2)	(30.5–82.9)	(−4.0–19.8%)		(20.6–79.3)	(41.3–90.6)	(−10.9–100.6%)		(33.5–39.9)
External rotators	34.8 ± 7.7	29.5 ± 9.2	−16.0 ± 13.8%	0.250	27.6 ± 2.9	37.1 ± 2.1	35.3 ± 11.8%	0.063	32.0
	(23.4–39.4)	(18.4–39.6)	(−30.4–2.2%)		(23.2–30.6)	(34.9–39.3)	(20.1–50.6%)		(29.9–34.2)
Elbow									
Flexors	52.0 ± 9.4	56.1 ± 10.0	8.4 ± 10.9%	0.375	63.1 ± 6.9	61.9 ± 8.6	−0.8 ± 19.8%	0.625	39.0
	(43.1–63.3)	(46.1–67.4)	(−3.0–20.2%)		(54.0–72.0)	(50.3–71.4)	(−21.2–32.3%)		(37.7–39.0)
Extensors	30.6 ± 6.3	36.7 ± 16.1	19.4 ± 37.8%	0.625	39.8 ± 20.7	45.0 ± 23.2	20.4 ± 26.5%	0.313	30.6
	(23.6–36.4)	(26.3–60.7)	(−25.5–66.9%)		(4.7–58.4)	(6.9–65.4)	(−20.8–45.4%)		(28.1–33.1)

## Supplementary Materials

Supplementary Materials Manual wheelchair cardiorespiratory fitness training programs questionnaire: questionnaire on satisfaction and perceived benefits following the completion of the training programs.
