# Supplementary material for: Feasibility, Safety, and Preliminary Effectiveness of a Home-Based Self-Managed High-Intensity Interval Training Program Offered to Long-Term Manual Wheelchair Users
Source: Rehabil Res Pract. 2018 May 17;2018:8209360. doi: 10.1155/2018/8209360 (PMC5985105; doi:10.1155/2018/8209360)
Supplement: Supplementary Materials — Manual wheelchair cardiorespiratory fitness training programs questionnaire: questionnaire on satisfaction and perceived benefits following the completion of the training programs. [file 8209360.f1.pdf]

Manual wheelchair cardiorespiratory fitness training programs
Satisfaction questionnaire following participation in the training program

General satisfaction

After reading each of the following statements, please indicate your level of agreement or disagreement.

1. In general, I am satisfied with the cardiorespiratory wheelchair training program that I recently completed at home with indirect supervision.

1	2	3	4	5	6	7
**Completely disagree**						**Completely agree**

2. I would recommend the training program that I recently completed to other people with a spinal cord injury.

1	2	3	4	5	6	7
**Completely disagree**						**Completely agree**

Feasibility of the training program

3. The total duration of the training program, which was spread over a period of 6 weeks, was not too restrictive considering my availability.

1	2	3	4	5	6	7
**Completely disagree**						**Completely agree**

4. The number of training sessions, which was 3 sessions per week, was adequate considering my availability.

1	2	3	4	5	6	7
**Completely disagree**						**Completely agree**

5. The duration of each training session, which was approximately 40 minutes, was adequate considering my availability.

1	2	3	4	5	6	7
**Completely disagree**						**Completely agree**

6. An initial home visit from a physiotherapist and weekly telephone follow-ups were adequate.

1	2	3	4	5	6	7
**Completely disagree**						**Completely agree**

7. I was able to reach the recommended levels of exertion for the training program I was assigned to.

1	2	3	4	5	6	7
**Completely disagree**						**Completely agree**

Perceived benefits for my health

8. At the end of the training program, I noticed general improvement in my health.

1	2	3	4	5	6	7
Significant deterioration	Moderate deterioration	Slight deterioration	No change	Slight improvement	Moderate improvement	Significant improvement

9. At the end of the training program, I noticed a change in my cardiorespiratory fitness level.

1	2	3	4	5	6	7
Significant deterioration	Moderate deterioration	Slight deterioration	No change	Slight improvement	Moderate improvement	Significant improvement

10. At the end of the training program, I noticed a change in my arm muscle strength.

1	2	3	4	5	6	7
Significant deterioration	Moderate deterioration	Slight deterioration	No change	Slight improvement	Moderate improvement	Significant improvement

11. At the end of the training program, I noticed a change in my overall endurance level during my daily activities.

1	2	3	4	5	6	7
Significant deterioration	Moderate deterioration	Slight deterioration	No change	Slight improvement	Moderate improvement	Significant improvement

12. At the end of the training program, I noticed a change in the performance of my daily activities.

1	2	3	4	5	6	7
Significant deterioration	Moderate deterioration	Slight deterioration	No change	Slight improvement	Moderate improvement	Significant improvement

13. At the end of the training program, I noticed a change in my sleep habits.

1	2	3	4	5	6	7
Significant deterioration	Moderate deterioration	Slight deterioration	No change	Slight improvement	Moderate improvement	Significant improvement

Perceived risks during training

14. I was afraid of reaching exertion levels that would exceed my capacity.

1	2	3	4	5	6	7
**Completely disagree**						**Completely agree**

15. I was afraid of causing or exacerbating musculoskeletal pain in my arms (i.e., wrists, elbows, or shoulders).

1	2	3	4	5	6	7
**Completely disagree**						**Completely agree**

16. I was afraid of losing my balance or falling.

1	2	3	4	5	6	7
**Completely disagree**						**Completely agree**

Motivation to continue engaging in physical activity

17. At the end of the wheelchair cardiorespiratory training program, I intend to continue with the training program that I started.

1	2	3	4	5	6	7
**Completely disagree**						**Completely agree**

Comments

18. If you have any general or specific comments to share with us, please let us know.

The research team would like to thank you for taking the time to complete this questionnaire.
